# Hernia Reduced by Elastic Bandage

**Published:** 1879-09

**Authors:** R. Tilley

**Affiliations:** Chicago


ARTICLE X.

*Hernia Reduced by Elastic Bandage.*

For the June No., 1878, of the JOURNAL AND EXAMINER, I remember abstracting from *L'Année Médicale* a summary of two cases of intractible hernia reduced by means of Esmarch's bandage. As will be seen by reference to the above abstract, taxis and position and chloroform had all been faithfully tried by competent men to no purpose; whilst the hernia was reduced readily by the constant pressure of the elastic bandage.

Since the above was published, I have spoken with several physicians on the subject, but none of them had ever used the bandage or seen it used for this purpose; and Dr. F. H. Hamilton, in his extensive article on the question of hernia in the *Hospital Gazette*, June 7, 1879, does not mention the use of the rubber bandage, notwithstanding the fact that its successful application forms the most convincing argument towards the establishment of the theory which he has labored to establish.

Whilst visiting Geneva Lake, Wisconsin, last week, I became acquainted with Dr. Geo E. Catlin, who had just then under his care a case of right femoral hernia, which had existed for three days. Dr. C. had diligently used taxis and position and the administration of morphia, but without result, when he invited me to see the case with him.

The patient, a woman of forty-four years, had been previously troubled with the hernia, but the reduction had been effected with ease. On one occasion, when it seemed more obstinate than usual the reduction was effected by a dose of morphia and the elevation of the hips. She was at the time troubled with other afflictions, which appeared to have no connection with the hernia. The tumor in question was hard and about the size of a hen's egg. Considerable nausea was present, but no vomiting.

Anxious to try taxis still more effectively, we lowered the patient's head, elevated her hips with pillows and cushions, and one of us patiently drew the bowels towards the epigastric region, while the other manipulated the hernia. These efforts were as unsuccessful as the previous ones. Chloroform was then administered, and the efforts renewed without effect. I then asked Dr. C. to allow me to apply the rubber bandage, which I suggested he should bring with him. The bandage was applied and we left the patient with the hips elevated to return in the course of two hours. Finding himself detained by a case of labor, Dr. C. requested me to drive up and see the patient. The hernia was not reduced, but I also observed that the bandage was not applied as snugly as desirable — it was really too wide. I took it off and re-applied it, and had the satisfaction, in a quarter of an hour, of hearing an exclamation, Oh! from the patient, which was but the expression of pain which the hernia produced in returning to its place.

The bandage used was three inches wide, probably one two inches would have been more convenient. It was first laid over the crest of the ilium on the affected side, brought over the hypogastric region and round the back and overlapping the end brought down the groin straight over the tumor; it was then brought round the back of the thigh, and once more passed round the body to be brought again over the hernia and firmly secured.

It is certainly not necessary to advocate at any length the application of force so gentle, equable and constant, in preference to the rude, uneven and inconstant pressure which the most practiced hand can apply. The probability is that instead of being a last resort, after several failures, it will become the first measure adopted, and find a place in our regular text-books.

CHICAGO. R. TILLEY, M.D.

